# An urgent need for diagnostic tools to address global mpox public health emergencies

**DOI:** 10.1128/jcm.01321-24

**Published:** 2025-06-05

**Authors:** Benjamin M. Liu, Zhilong Yang

**Affiliations:** 1 Division of Pathology and Laboratory Medicine, Children’s National Hospital, Washington, DC, USA; 2 Department of Pathology, The George Washington University School of Medicine and Health Sciences 43989, Washington, DC, USA; 3 Department of Microbiology, Immunology and Tropical Medicine, The George Washington University School of Medicine and Health Sciences 43989, Washington, DC, USA; 4 Department of Veterinary Pathobiology, College of Veterinary Medicine & Biomedical Sciences, Texas A&M University 14736, College Station, Texas, USA; Vanderbilt University Medical Center, Nashville, Tennessee, USA

**Keywords:** mpox, monkeypox virus, diagnostic assays, PCR, point-of-care test, low- and middle-income countries

## Abstract

Monkeypox virus (MPXV) is the causative agent of mpox, a zoonosis formerly known as monkeypox. MPXV can be divided into clades I and II, which are further divided into subclades Ia, Ib, IIa, and IIb. Since May 2022, subclade IIb MPXV has rapidly spread outside Africa to more than 100 countries due to increased human-to-human transmission. Clade I is a more virulent MPXV endemic in Central Africa with up to 10% mortality in humans. Clade I has recently evolved into a novel subclade Ib and caused outbreaks in non-endemic neighboring countries and other continents. In response to mpox, the World Health Organization has declared Public Health Emergencies of International Concern in July 2022 (subclade IIb) and August 2024 (subclade Ib). The emergence and spread of the more virulent subclade Ib MPXV has caused a significant global public health threat, particularly in low- and middle-income countries (LMICs). The evolution of MPXV has outpaced the development of novel diagnostic assays, hampering the global response. There is an urgent need for additional diagnostic tools for the detection and surveillance of MPXV, especially subclade Ib MPXV, in LMICs. Herein, we provide the current epidemiology of mpox, analyze the diagnostic gaps for mpox, and evaluate the potential of additional detection strategies to be added to the suite of mpox assays. This commentary not only sheds light on the currently available diagnostic tools for mpox but also highlights the urgent need for additional diagnostic tools in response to the new global mpox public health threats.

## COMMENTARY

Mpox, caused by the monkeypox virus (MPXV), is a zoonotic disease formerly known as monkeypox. On 14 August 2024, the World Health Organization (WHO) declared the mpox outbreaks due to the emerging subclade Ib MPXV a Public Health Emergency of International Concern (PHEIC) (1). The novel subclade Ib MPXV was identified in 2024. It has enhanced human adaptation and has caused sustained human outbreaks. It is responsible for a growing number of mpox cases and deaths in Africa and for rapid spread to non-endemic neighboring countries and other continents, including Europe (Belgium, Germany, France, Sweden, and UK), Asia (India, China, Oman, Pakistan, and Thailand), and North America (Canada and the USA) (2–5). Of note, this is WHO’s second mpox-associated PHEIC determination in 2 years. There was an earlier PHEIC due to the milder subclade IIb MPXV that was announced in July 2022 and lifted in May 2023 (6). Facing the constantly evolving MPXV into a novel subclade Ib, it has been apparent that there are major gaps in diagnostic tool diversity and capacity in diagnosing, surveilling, and monitoring subclade Ib MPXV, especially in low- and middle-income countries (LMICs) (7–9). Herein, we provide an update on MPXV and mpox epidemiology, analyze currently available mpox tests, and evaluate potential resolutions to the diagnostic gaps.

## MPOX EPIDEMIOLOGY

MPXV, an enveloped, double-stranded DNA virus, belongs to the *Orthopoxvirus* (OPXV) genus within the *Poxviridae* family (10). In this family, there are over 80 species, including vaccinia virus (VACV) and variola virus (VARV; the agent causing smallpox) (10). Based on genetic sequences, MPXV can be divided into clades I and II (11). Clade I was originally identified in the Congo Basin and is associated with >10% of case fatality rate (CFR) (11). Before 2023, clade I was mainly transmitted from animals to humans with limited human-to-human spread. A new subclade, Ib, was identified in 2024. It has a lower CFR (~3%) and caused sustained human outbreaks in eastern Democratic Republic of the Congo (DRC) (3). Interestingly, the subclade Ib was found to have increased mutations consistent with induction by a human innate immune enzyme called APOBEC3 (Apolipoprotein B mRNA Editing Catalytic Polypeptide-like 3), indicating its increased adaptation to the human host (3). Clade II MPXV can be further classified into subclades IIa and IIb. Subclade IIa was found in West Africa (Nigeria, Liberia, Sierra Leone, and Côte d'Ivoire) and caused infection associated with approximately 1% mortality (12). In contrast, subclade IIb has a much lower mortality in immunocompetent individuals and is responsible for the 2022–2023 global mpox outbreaks (11).

**Fig 1:**
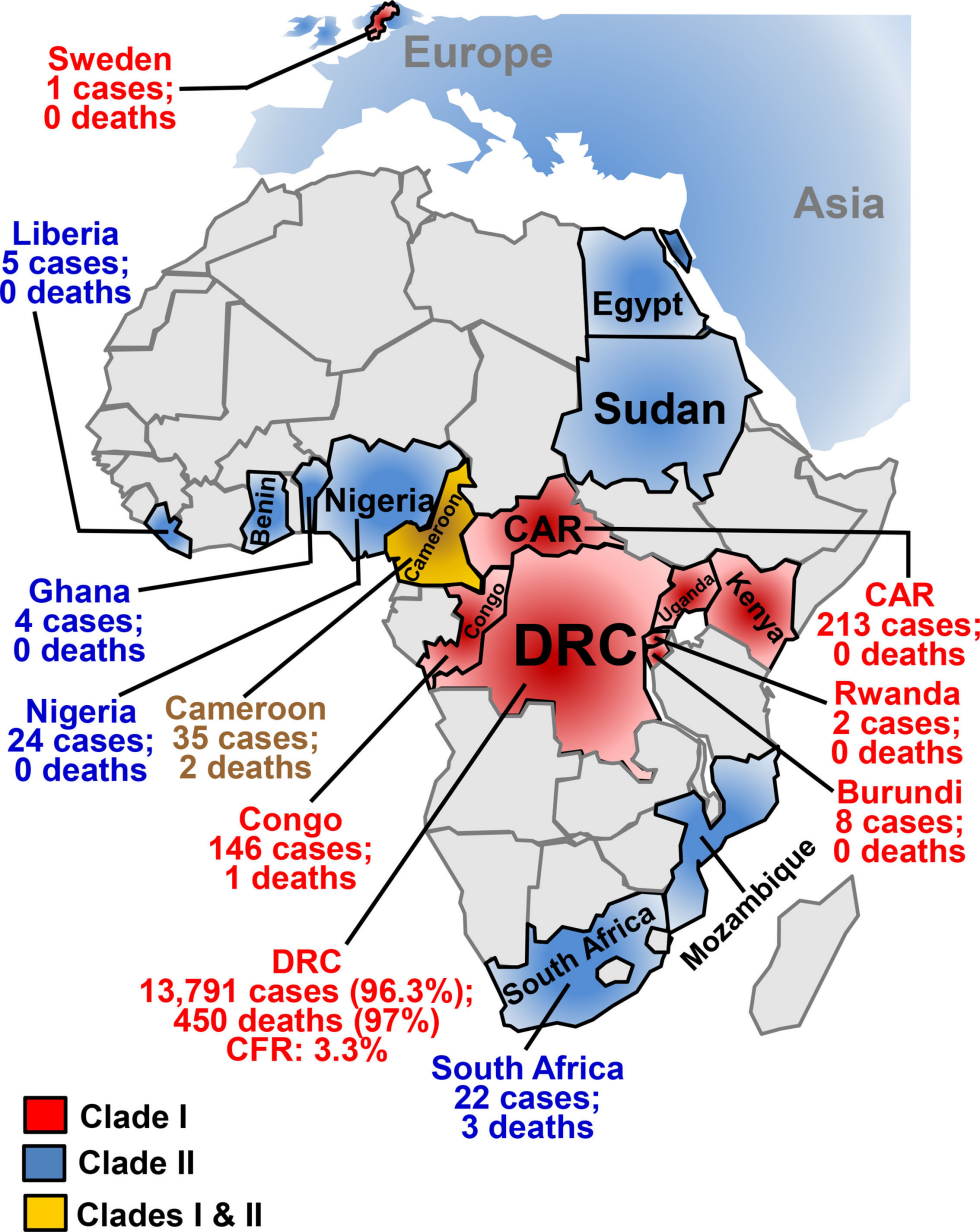
The spread of subclades Ib and IIb MPXV in Africa and other parts of the world as a global public health threat. Red-, blue-, and yellow-highlighted countries are those suffering from outbreaks due to clade I, clade II, and both clades, respectively, since the beginning of 2022. Reported mpox cases and deaths of 12 African countries between January and July 2024 were indicated, including seven red-highlighted countries (Congo, Democratic Republic of Congo [DRC], Central African Republic [CAR], Rwanda, Uganda, Kenya, and Burundi) with clade I, four blue-highlighted countries (Liberia, Ghana, Nigeria, and South Africa) with clade I, and yellow-highlighted Cameroon with both clades. On 15 August 2024, Sweden reported the first subclade Ib MPXV infection case. The map was drawn by the author.

While other parts of the world witnessed decreased incidence and spread of subclade IIb MPXV in 2024, Africa had a 160% increase in mpox cases and a 19% increase in mpox-associated mortality (Fig. 1) (13, 14). Most of these cases and deaths in Africa were due to clade I MPXV, which disproportionately affects women and young children (accounting for more than 40% of the infected population) (9, 13–15). Between January and July 2024, a total of 14,250 cases and 456 deaths of mpox were reported from 10 African countries, with an overall 3.2% CFR (Fig. 1) (13). Patients in the DRC infected with subclades Ia and Ib account for 96.3% of all cases and 97% of all deaths reported in this period (13). Clade I virus was also detected in Burundi, Cameroon, Central African Republic, Congo, Rwanda, Uganda, and Kenya (Fig. 1) (13). As of January 2025, subclade Ib MPXV has been reported in multiple countries outside Africa, for example, Germany, India, Sweden, Thailand, UK, and the USA (2, 4, 5).

## GAPS IN THE ACCESSIBILITY OF DIAGNOSTIC TESTS IN THE GLOBAL RESPONSE TO MPOX OUTBREAKS

Since the declaration of the 2024 mpox PHEIC, progress has been made in boosting the response to subclade Ib MPXV, including ramping up the availability of mpox testing, improving access to vaccines, reducing the spread of disease, and supporting the affected communities (8, 9). However, inequity in globalizing testing and insufficient funding of mpox response measures are hampering the global response for two reasons (8, 9). First, there is limited diagnostic capacity for clade I MPXV in Central Africa (9, 15). An inadequate global supply of diagnostic tests in this region has led to a lack of access to timely detection and diagnosis of mpox. Second, there is a lack of rapid diagnostic tests for mpox, which are critical for timely detection and containment of outbreaks, to stop ongoing transmission and to monitor and orient the response to the epidemic (16, 17). The utilization of the ring vaccination strategy to curtail mpox outbreaks in Africa, i.e., vaccinating close contacts of confirmed cases, requires timely detection of index cases and swift and thorough surveillance and epidemiologic case investigation (18, 19). Shortages of high-sensitivity and point-of-care (POC) rapid diagnostic tools leave most suspected cases in outbreaks unconfirmed and delay timely detection and vaccination, especially in LMICs.

## LIMITATIONS OF CURRENTLY AVAILABLE TESTS FOR MPOX

There are at least six major limitations of currently available mpox tests for the diagnosis, surveillance, and monitoring of subclade Ib and IIb MPXV, which hamper the global response to MPXV in LMICs and high-income countries. Some of these limitations are related to the evolution and spread of MPXV. The rapid global spread of subclade IIb MPXV and the emergence of novel subclade Ib in Africa suggest that mpox has adapted to human hosts (3, 11). The evolution of MPXV in different geographical areas has outpaced the development of novel diagnostic assays for the detection of novel MPXV strains with new genome sequences or the differentiation of subclade Ib from subclade IIb. Clade identification is important due to differences in virulence and CFR of the clades, and because clade I MPXV, but not low-virulence clade II, is a select agent.

First, mpox real-time PCRs with non-VARV OPXV (NVO) and clade II-specific MPXV targets (Table 1), which are used by US Centers for Disease Control and Prevention (CDC) and some commercial reference labs in the United States, have played important roles in handling the 2022–2023 subclade IIb mpox outbreak (8, 11, 20). However, the NVO PCR does not distinguish subclade Ib from clade II MPXV. In response to the 2024 subclade Ib mpox PHEIC, an algorithm for the interpretation of mpox real-time PCRs that use NVO and clade II MPXV PCR targets (21) should be updated to include a scenario of subclade Ib MPXV (Table 2). Specifically, when NVO targets are positive, but clade II-specific MPXV targets are negative, a presumptive diagnosis or suspicion for clade I MPXV should be considered (Table 2). The samples should be referred to a public health laboratory for confirmation of clade I (Table 2), which may prolong the turnaround time (22, 23). In addition, there may be other possible explanations for positive NVO targets and negative clade II-specific MPXV targets, for example, mutations in clade II-specific MPXV targets (Table 2).

**TABLE 1:** Currently available and potential diagnostic tools for mpox^*a*^

Diagnostic tools^*b*^	Methods	Pros	Cons
Non-VARV OPXV (NVO) NAAT	Real-time PCR Isothermal NAAT, for example, LAMP	US CDC’s NVO PCR is FDA-cleared Targeting a conserved gene with little impact from mutations A positive result provides a presumptive mpox diagnosis Isothermal NAATs have their independence from expensive instrumentation, rapid TAT, ease of use, and POC application potential	Fails to differentiate OPXVs, let alone MPXV clades VACV-derived live smallpox vaccine may cause false positives Atypical mpox or swabbing rashes due to other co-infected pathogens may cause false negatives Relatively expensive
Clade-specific MPXV NAAT	Real-time PCR, for example, TNF receptor (*TNFR*) gene-targeted PCR Isothermal NAAT, for example, LAMP CRISPR-Cas-based nucleic acid detection technology	*TNFR*-targeted PCR can differentially detect clade II MPXV; some LRN reference labs detect clade I MPXV, a select agent Per CDC, NVO and clade-specific tests should be used together. Real-time PCRs are the gold standard of diagnostic method for mpox Coupled with isothermal NAATs, CRISPR-Cas-based diagnostics have rapid TAT, ease of use, and POC application potential, with independence from expensive instrumentation	Tests for clade I MPXV are unavailable in non-LRN labs Test efficacy is affected by potential MPXV genomic deletions Varied sensitivity/accuracy Relatively expensive No FDA-approved isothermal NAATs or CRISPR-Cas-based diagnostics for mpox
Sample-to-answer NAAT: Cepheid Xpert Mpox test	Real-time PCR	FDA EUA in US Clade II-specific detection The POC test yields clinically actionable results TAT = 36 min	Cannot report any results about clade I MPXV Test efficacy may be affected by potential MPXV genomic deletions Need specific instruments
Sequencing assays	Sanger sequencing Next-generation sequencing	Can differentiate MPXV clades Can detect known or novel MPXV mutations Epidemiology investigation or genomic surveillance	Not clinically actionable due to long TAT Limited accessibility in low-resource settings Expensive
Rapid antigen test	Enzyme-linked immunosorbent assays (ELISA) Lateral flow immunoassays	Potentially can enable rapid or POC test due to short TAT Potentially can enable early diagnosis after mpox onset Low cost	Limited data on clinical accuracy Cannot rule out infection based on negative results May have lower sensitivity than PCR There are no antigen tests recommended for the purpose of mpox diagnostics
Viral culture	Traditional viral culture Shell vial culture	Gold standard of detection Accepts a broad range of sample sources Viral isolation allows for further virology studies	Clinical labs should not cultivate samples suspected of clade I MPXV, a select agent CPE fails to differentiate OPXVs or MPXV clades Less sensitive and slower than PCR Requires special expertise
Antibodies (IgG and/or IgM) detection	ELISA Lateral flow immunoassays Multiplex immunoassays	Useful for understanding population-level exposures Utility in using antibody titers to correlate with MPXV immunity Fast TAT Inexpensive	Time lag in antibody development Antibody detection only indicates MPXV exposure May fail to differentiate MPXV clades or other OPXV Low utility for acute illness There are no antibody tests recommended for the purpose of mpox diagnostics

^*a*^ Cas, CRISPR-associated; CPE, cytopathic effect; CRISPR, clustered regularly interspaced short palindromic repeats; EUA, Emergency Use Authorization; FDA, US Food and Drug Administration; LAMP, loop-mediated isothermal amplification; LRN, Laboratory Response Network; NAAT, nucleic acid amplification test; PCR, polymerase chain reaction; POC, point of care; TAT, turnaround time; and TNF, tumor necrosis factor.

^*b*^ Seven categories of diagnostic tools are presented in this Table. Representative methods, pros and cons of the same category of diagnostic tools are shown with bullet lists with the same symbols (either dots or squares) on the corresponding rows.

**TABLE 2:** Interpretation algorithm of diagnostic real-time PCRs for mpox^*a*^

Non-VARV OPXV PCR result	Clade II MPXV-specific PCR result	Interpretation	Scenarios/explanations^*b*^	Troubleshooting actions^*b*^
Negative	Negative	MPXV not detected	MPXV DNA is not present in the tested sample	Negative results must be evaluated with a combination of patient history, clinical observations, and epidemiological information If there is still suspicion of MPXV infection, consider collecting and testing another specimen
Positive	Positive	Clade II MPXV detected	Clade II MPXV DNA is present in the tested sample	Clinical correlation with patient history, clinical observations and other diagnostic test results is necessary to determine MPXV infection status If there is a suspicion of false positive results, follow general procedures for troubleshooting
Negative	Positive	Clade II MPXV detected	Low level of viral DNA in the sample may cause the discrepant results of both PCRs	Check *C*_*T*_ values and amplification curve of the positive PCRs Repeat DNA extraction and the PCRs
Positive	Negative	OPXV detected, but clade II MPXV not detected	Other OPXV DNA may be present A presumptive diagnosis or suspicion for clade I MPXV should be considered	The sample should be referred to a public health laboratory for confirmation of clade I MPXV or other OPXV
			This pattern may indicate a mutation(s) in the MPXV target gene	Use another orthogonal clade II MPXV-specific PCR targeting a different gene for repeat testing Sequencing assay may be considered to confirm the existence of a mutation(s) in the affected PCR
			Low level of viral load in the sample may cause the discrepant results of both PCRs	Check *C*_*T*_ values and amplification curve of the positive PCRs Repeat DNA extraction and the PCRs

^*a*^ C_*T*_, cycle threshold; PCR, polymerase chain reaction.

^*b*^ Troubleshooting actions for the corresponding scenarios/explanations are shown with bullet lists with the same symbols (either dots or squares) on the corresponding rows.

Second, the WHO has listed the cobas MPXV assay (Roche Molecular Systems, Pleasanton, CA, USA) and the Alinity m MPXV assay (Abbott Molecular, Des Plaines, IL, USA) under Emergency Use Listing (EUL) to cope with mpox outbreaks in Central Africa. Both assays are suitable for a centralized reference laboratory setting with trained clinical laboratory personnel with proficiency in PCR techniques and *in vitro* diagnostic procedures (8). However, these high-throughput mpox tests cannot differentiate between clades I and II MPXV. The cobas MPXV assay targets MPXV genes F3L and B21R/B22R, and the Alinity m MPXV assay targets MPXV genes B7R and J2R; neither differentiates clades I and II MPXV (24, 25).

Third, in the US CDC guidelines, testing for MPXV infections is only indicated for symptomatic patients with a rash that is typical for mpox (26). Lesion swabs are the only sample source recommended for mpox testing by the WHO and the US CDC (11). Accordingly, many commercial reference laboratories (for example, ARUP, Quest Diagnostics, Labcorp, and Mayo Clinic) offering mpox tests in the US only accept lesion swabs for testing (27–31). However, there are several limitations to using lesion swabs alone for mpox testing. Although skin lesion samples are confirmed to give high yield in MPXV PCR testing for typical symptomatic patients (32–36), mpox cases that have few or atypical skin lesions may be detected poorly by skin swabs. This is also true for patients with asynchronous skin lesions, mucosal involvement (for example, oral and tonsillar ulcers, pharyngitis, epiglottitis, proctitis, or ulcerations), or conjunctival involvement (11). Besides, lesion swab-based mpox PCR assay can also cause false-positive results in pregnant women and pediatric cases due to non-specific amplifications (11, 37).

Fourth, there are a limited number of rapid mpox tests, for example, molecular POC tests, that can be deployed to remote regions in mpox-endemic countries (8, 9). Of note, Xpert Mpox (Cepheid, Sunnyvale, CA, USA) is the only POC molecular test under WHO EUL (38) (Table 1). This test is a US Food and Drug Administration (FDA) Emergency Use Authorized (EUA) real-time PCR test intended for the qualitative detection of clade II MPXV and NVO targets in human lesion swab specimens from individuals suspected of mpox by their healthcare provider in the POC setting (38). The interpretation algorithm for NVO and clade II MPXV PCRs (Table 2) also applies to the Cepheid Xpert Mpox test. A presumptive diagnosis or suspicion for clade I MPXV should be considered when NVO targets are positive, but clade II-specific MPXV targets are negative (Table 2). Xpert Mpox test exhibited 100% inclusivity to clade II MPXV sequences but no significant match with molluscum contagiosum virus (38), a poxvirus commonly responsible for self-limited infectious dermatosis among pediatric populations (11).

Furthermore, there is a lack of MPXV antigen rapid diagnostic tests (RDTs) (Table 1) that could enable rapid diagnosis of acute infection by detecting viral proteins at the POC. RDTs that detect viral antigens often have lower sensitivity than nucleic acid amplification tests (NAATs), and in some cases, they cannot be used to rule out infection with a negative antigen test. In a recent systematic search by the Africa CDC Diagnostic Advisory Committee, no antigen RDTs have exhibited the minimum sensitivity requirement for mpox testing (39).

Finally, like other DNA viruses that accumulate antiviral drug resistance (ADR) mutations, a significant concern for the current antiviral therapy (i.e., brincidofovir/cidofovir and tecovirimat) for mpox is the emergence of drug resistance (11). MPXV resistance to tecovirimat has been reported in a small patient population with advanced HIV receiving treatment for a duration of weeks to months (40). Tecovirimat targets the MPXV F13L gene homolog, whereas single amino acid changes in F13 are believed to lead to resistance to tecovirimat (40). In addition, mutations A314T and A684V in the VACV DNA polymerase (E9L) gene, which confer cidofovir resistance, have been identified in VACV using an *in vitro* selection model, though cidofovir resistance has not been reported in clinical cases (11). Genomic surveillance to monitor the evolution of ADR MPXV variants is warranted (11). However, traditional sequencing-based methods cannot provide clinically actionable results due to their long turnaround time, high cost, requirement for skilled technicians, and limited accessibility in LMICs (41) (Table 1).

## POTENTIAL RESOLUTIONS FOR DIVERSIFYING DIAGNOSTIC TOOLS FOR MPOX

There are five potential resolutions on the diagnostic gaps for mpox.

First, it is important to design and implement subclade-specific primers and probes as well as real-time PCR assays for the detection of subclade Ib MPXV (Table 1). It is challenging to develop robust, clade- or subclade-specific MPXV assays due to the fact that there is a high sequence identity between clades I and II MPXV (>99%), as well as with other OPXV (>90%) (11, 42, 43). Therefore, insertions and deletions in the genome of DNA viruses can be identified as target regions to discriminate between the different strains and clades (42, 43). Following this idea, Schuele et al. (44) developed a real-time PCR assay for the detection of subclade Ib MPXV. The limit of detection of the novel assay for subclade Ib and clade II MPXV was 6.6 and 59.9 copies/reaction, respectively (44). The assay can differentiate clades I and II MPXV (44). The assay determined 100% (82/82) of the MPXV positive samples as subclade Ib, which were collected during the mpox outbreak in the South Kivu region of DRC between September 2023 and May 2024 (44). Therefore, the novel subclade Ib-specific real-time PCR assay exhibited high sensitivity and specificity (44).

Second, validating different specimen types is necessary for mpox tests. While lesion sampling is helpful for symptomatic mpox cases, choosing other symptomatic sites or sample types may provide higher diagnostic yield for mpox testing or be helpful for early detection of mpox before the development of skin lesions (7). For example, rectal swabs may have high yield for MPXV-associated proctitis, upper respiratory specimens (for example, oral swab, nasopharyngeal swab, and saliva) for cases with oropharyngeal involvement, and blood samples (for example, whole blood, plasma, and sera) for systemic infections (7, 11, 32–35).

Third, with POC technologies becoming increasingly available, it is essential to develop novel, rapid, and inexpensive POC tests for MPXV, including POC molecular tests with a diversified choice of NAAT (20, 45–47). Compared with PCR, which requires a thermocycler and detection instruments, isothermal NAATs, such as loop-mediated isothermal amplification (LAMP) and recombinase polymerase amplification (RPA) assays, are attractive diagnostic tools for MPXV tests due to their independence from expensive instrumentation, rapid turnaround times, ease of use, and POC application potential (7, 45) (Table 1). LAMP-based molecular assays recognize the target DNA by six distinct sequences initially and four distinct sequences afterward, thereby ensuring high specificity and selectivity (45). LAMP has high tolerance to well-known PCR inhibitors (45). LAMP-based assays have been developed as a rapid, reliable, and inexpensive POC test in LMICs (45, 46). For instance, a LAMP-based assay for tuberculosis has been recommended as the diagnostic test for active pulmonary tuberculosis in the primary care settings and added to the first WHO List of Essential *In Vitro* Diagnostics (45). In response to mpox outbreaks, Feng and colleagues (48) developed a LAMP-based mpox assay to enable rapid, sensitive detection of MPXV DNA in primary hospitals and rural areas. Li and colleagues (49) developed an extraction-free colorimetric LAMP assay enabling rapid and sensitive detection and differentiation of OPXV and MPXV, which represents a valuable tool for rapid and scalable diagnosis and surveillance of OPXV and MPXV, especially in low-resource settings. Moreover, Davi and colleagues (50) developed an RPA-based assay enabling rapid detection of MPXV DNA at low-resource settings using a solar-powered mobile suitcase laboratory.

Clustered regularly interspaced short palindromic repeat (CRISPR) and CRISPR-associated (Cas) technology, especially those using Cas12a, Cas13a, and Cas14 with collateral cleavage activities, makes it possible to develop rapid, instrument-free, and field-deployable NAATs for MPXV (45, 46) (Table 1). Low and colleagues (51) developed a portable isothermal amplification CRISPR-Cas12a-based POC assay for sensitive detection of MPXV DNA. Hirano and colleagues (52) recently developed a device-free, sustainable, and field-adapted CRISPR-Cas3-based diagnostics for highly sensitive and rapid detection of multiple subclades of MPXV (Ia, Ib, and IIb). Following the ASSURED (Affordable, Sensitive, Specific, User-friendly, Rapid/Robust, Equipment-free, Deliverable) principles for diagnostics development (53), both isothermal NAATs (for example, LAMP) and CRISPR-Cas systems have demonstrated their potential for the development of POC testing for MPXV.

Fourth, next-generation sequencing (NGS) and whole-genome sequencing (WGS) have demonstrated their utility in poxvirus genomics and global genomic surveillance of MPXV (54, 55) (Table 1). Genomic surveillance using sequencing-based methods may be useful to monitor the evolution of ADR-associated MPXV variants. To overcome the limitations of sequencing-based technologies (for example, long turnaround time), Nanopore long-read sequencing-based WGS shows promise to be developed into a portable near-POC rapid sequencing assay for field study (7). Amplicon-based targeted NGS may solve the sensitivity issue of sequencing-based technologies in the direct detection of AMR mutants from clinical samples. Kwasiborski and colleagues (56) adopted Nanopore MinION technology to develop amplicon-based sequencing and shotgun metagenomic sequencing assays to detect and analyze the whole-genome sequence of MPXV directly from clinical specimens during the 2022 epidemic. Using Nanopore MinION real-time long-read sequencing, Vandenbogaert and colleagues (57) evaluated different polishing tools on MinION-sequenced genomes and analyzed MPXV genomes originating from lesion samples in a remote area of the Central African Republic.

Finally, given that MPXV antigen RDTs are more accessible and cheaper than molecular tests, more research is warranted to understand whether and how well antigen tests can be used and accumulate more data to determine their clinical utility (58) (Table 1). There are RDT lateral flow immunoassays for detecting MPXV antigen that have received Conformité Européenne *in vitro* diagnostic product approval (59), whose clinical utility warranted further verification. To overcome the low sensitivity issue of antigen RDTs, choosing single-molecule antigen detection using nanopores may enhance the sensitivity of antigen detection (60). Employing this technology, Cai and colleagues (60) developed a nanopore-sensing strategy empowering rapid and sensitive detection of MPXV antigen (A29 protein), which shows potential for the application in POC settings.

Of note, serologic testing has little role in the diagnosis of acute mpox. MPXV IgG positive results only indicate exposure to the virus but not current infections (Table 1). There is a time lag (approximately 10–20 days) in antibody development and low utility for acute illness. More importantly, serologic methods cannot differentiate MPXV clades and even different OPXV members. Serologic testing may be useful for understanding population-level exposures and predicting protective immunity by correlating antibody titers with neutralizing antibodies (Table 1) (20).

## CONCLUSIONS AND PERSPECTIVES

The evolution and emergence of subclade Ib MPXV in Africa and other continents has not only constituted a major threat to the local population with low immunity but also caused global public health concerns. Although real-time PCR assays are useful diagnostic tools for MPXV infections, there are unmet gaps in the development of rapid, sensitive, and specific assays for subclade Ib and other MPXV, especially in LMICs. Most of the real-time PCRs are only compatible with centralized lab settings. There is only one FDA EUA POC molecular test. None of the commercially available mpox testing can definitively detect or report clade I MPXV, a select agent. This commentary calls for accelerated efforts for developing novel diagnostic assays for mpox and related clinical utility studies. Among different potential testing strategies, POC molecular tests using isothermal NAAT and CRISPR-Cas systems are promising ones to be developed. Assay developers, companies, research labs, and public health sectors should work together to develop a diversity of rapid MPXV testing reagents and methods to address new global mpox public health emergencies.
